# Bungeanum Improves Cognitive Dysfunction and Neurological Deficits in D-Galactose-Induced Aging Mice *via* Activating PI3K/Akt/Nrf2 Signaling Pathway

**DOI:** 10.3389/fphar.2020.00071

**Published:** 2020-02-25

**Authors:** Meihuan Zhao, Xueqian Tang, Daoying Gong, Peng Xia, Fushun Wang, Shijun Xu

**Affiliations:** ^1^ School of Pharmacy, Chengdu University of Traditional Chinese Medicine, Chengdu, China; ^2^ Institute of Material Medica Integration and Transformation for Brain Disorders, Chengdu University of Traditional Chinese Medicine, Chengdu, China; ^3^ Department of Pathology, Hospital of Chengdu University of Traditional Chinese Medicine, Chengdu, China; ^4^ Institute of Brain and Psychological Sciences, Sichuan Normal University, Chengdu, China

**Keywords:** *Zanthoxylum bungeanum* Maxim., cognitive dysfunction, fear memory, neurological deficits, oxidative stress, PI3K/AKT/Nrf2 signaling

## Abstract

*Zanthoxylum bungeanum* Maxim (Rutaceae), a popular condiment and dietetic herbal medicine, has been used traditionally in the treatment of forgetfulness, as recorded in *Shen Nong's Herbal Medicine*, an old Chinese medicine book. To explore effects and potential mechanisms of it, extracts of *Z. bungeanum* through water (WEZ), volatile oil (VOZ), petroleum ether (PEZ), and methylene chloride (MCZ) were used to treat the memory loss induced in D-galactose-induced aging mice. The impaired memory was significantly alleviated after WEZ and VOZ extract treatment. WEZ and VOZ extracts also prevented D-galactose-induced hippocampal neuron damage. In addition, WEZ and VOZ extracts upregulated nuclear factor erythroid 2-related factor 2 (Nrf2) and heme oxygenase 1 (HO-1), which suggests that the effects of WEZ and VOZ extracts on oxidative stress and apoptosis might be involved in the cognitive dysfunctions. Furthermore, WEZ and VOZ extracts enhanced the activation of phosphoinositide 3-kinase (PI3K)/protein kinase B (Akt), which suggests that *Z. bungeanum* has an appreciable therapeutic effect on learning and memory disabilities, and its mechanism may be related to activate PI3K/Akt signaling pathway. Collectively, our study suggested that *Z. bungeanum* extracts are promising agents for prevention of aging-related cognitive dysfunction and neurological deficits.

## Introduction

Aging is a major cause for inducing cognitive impairment in humans, and it is a major risk factor for neurodegenerative diseases that are associated with neurological deficits (Liang et al., 2019). Many prevalent neurological disorders are closely related to aging (Juan and Adlard, 2019). The mitochondrial free-radical theory suggests that aging is caused by oxidative damage to macromolecules caused by mitochondrial reactive oxygen species (ROS) (Barja, 2019). Thus, preventing oxidative stress-induced neuronal degeneration might be crucial to prevent the aging process and its associated neurodegenerative diseases such as Alzheimer's disease (AD). D-galactose is a physiological nutrient that is involved in glucose metabolism, but its excessive accumulation produces redundant ROS formation and decreases endogenous antioxidant enzyme activity, cognitive dysfunction, and neurological deficits in rodents (Hao et al., 2014; Sadigheteghad et al., 2017). Consequently, D-galactose-induced aging mice were used to mimic aging brain pathology in this present study.

Nuclear factor erythroid 2-related factor 2 (Nrf2) is a master regulator for regulating oxidative stress *via* the activation of transcription, which in turn increases the secretion of antioxidant enzyme and regulates antioxidant gene expressions, such as heme oxygenase 1 (HO-1) (Gong et al., 2019). When oxidative stress or damage increased in aging mammals, Nrf2 knockout mice exhibited severe susceptibility to oxidative damage and decreased expression of Nrf2 (Zhang et al., 2017), indicating that Nrf2 has neuroprotective effects. In addition, the activation of phosphoinositide 3-kinase (PI3K)/protein kinase B (Akt) signaling pathway promotes the translocation of Nrf2 from the cytoplasm to the nucleus to facilitate the transcription of detoxification enzyme and antioxidant enzyme protein gene (Zhuang et al., 2019). Some drugs were reported to activate PI3K/Akt signaling pathway and promote the transcription of Nrf2 to relieve cognitive impairment and neurological deficits (Liang et al., 2019). Thus, the Nrf2 has been regarded as a potential therapeutic target for the treatment of neurodegenerative diseases.

**Figure 1:**
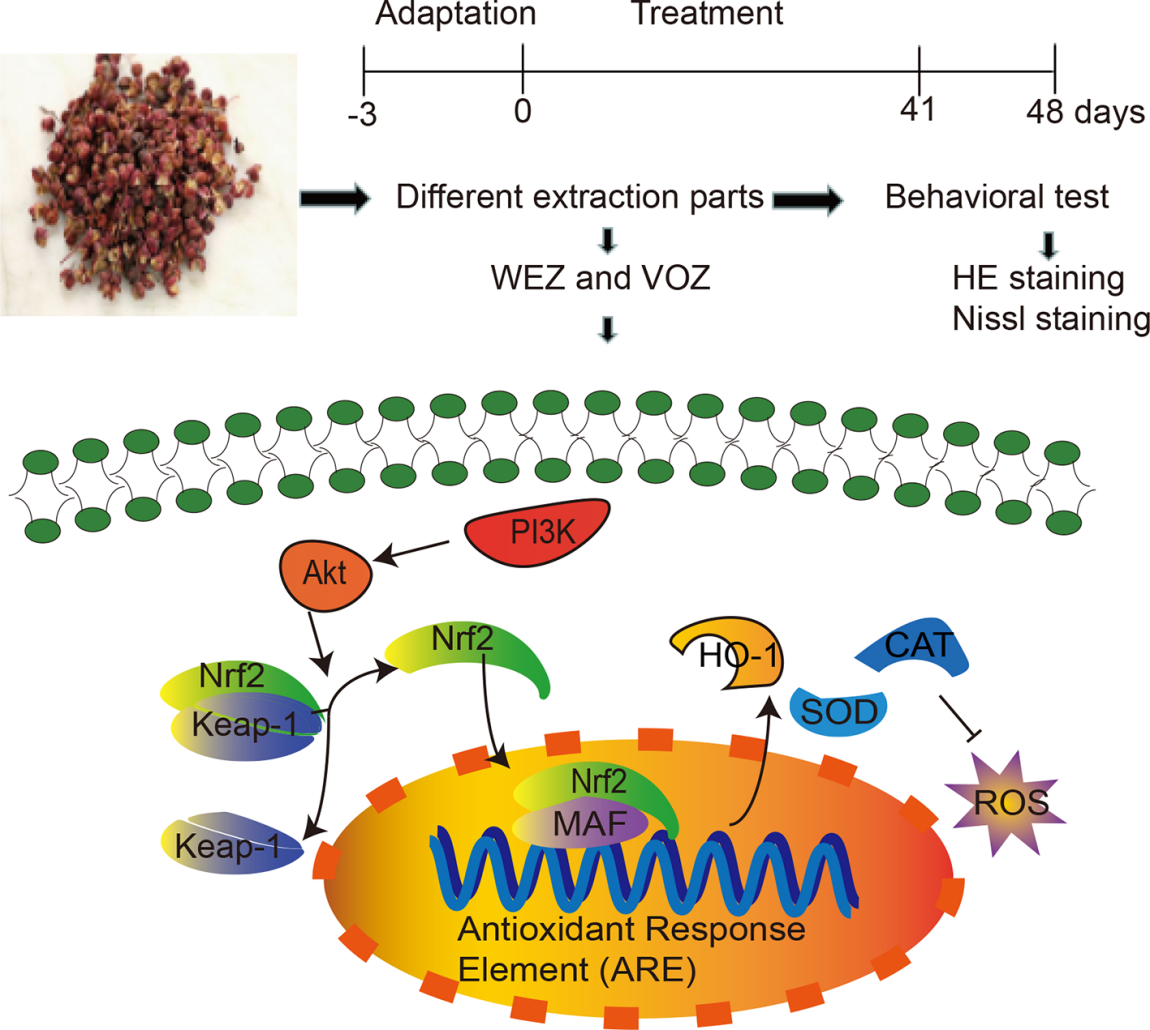
A detailed illustration of the experiment.

*Zanthoxylum bungeanum* Maxim. (*Z. bungeanum*), an exceedingly popular spice with pleasant pungent sensation, is widely used in East and Southeast Asia (Menozzi-Smarrito et al., 2009). *Z. bungeanum* is also involved in more than 30 traditional medical prescriptions in the treatment of various diseases, including forgetfulness, poor appetite, toothache, abdominal pain, vomiting, diarrhea, ascariasis, and trauma (Zhang et al., 2017; Deng et al., 2019). Many compositions in *Z. bungeanum* are reported to have anti-inflammatory, antitumor, antibacterial, antioxidant, and neuroprotective properties (Zhang et al., 2017; Chen et al., 2018). Especially, the antioxidant activity of *Z. bungeanum* contained a variety of bioactive polyphenols, which might activate the immune system and prevent neuronal impairment in neurophysiology (Deng et al., 2019). However, the mechanisms are still unclear. In this study, we investigated the neuroprotective mechanisms underlying *Z. bungeanum* against D-galactose-induced cognitive dysfunctions. Our results show that *Z. bungeanum* treatment attenuates D-galactose-induced cognitive impairment, oxidative stress, and apoptosis possibly through PI3K/Akt/Nrf2 signaling pathway. A detailed illustration of the study is shown in **Figure 1**.

## Materials and Methods

### Animals

A total of 70 male adult Kunming mice (18–22 g; Dashuo Experimental Animal Co., Ltd., Chengdu, China) were used in this work. All mice were housed in a standard 12-hour light/dark cycle with free access to water and food. The mice were acclimated to the animal facility for 3 days before conducting the experiments. All experiments were approved by the Ethics Committee for Animal Experiments of the Institute of Material Medica Integration and Transformation for Brain Disorders in Chengdu University of Chinese Medicine and accorded with the National Institutes of Health Guide for the Care and Use of Laboratory Animals.

### Materials and Reagents

*Z. bungeanum* Maxim. (batch number 20180802) was purchased from Hehuachi tradition herbal medicine market, Chengdu, China. Mesylate dihydroergotoxine tablets (batch number 7K893T) were purchased from Tianjin Huajin Pharmaceutical Co., Ltd., Tianjin, China. D-galactose (batch number 2017060101) was purchased from Chengdu Cologne Chemical Co., Ltd., Chengdu, China. Superoxide dismutase (SOD) kit, malondialdehyde (MDA) kit, glutathione (GSH) kit, catalase (CAT) kit, and nuclear protein extraction kit were obtained from Nanjing Jiancheng Bioengineering Institute, Nanjing, China (batch number 20180605, 20180611, 20190718, 20190719, and 20190722, respectively). Primary antibodies, including PI3K (Cat no.: 4257S), AKT (Catno.: 4685S), *p*-PI3K (Cat no.: 4685S), *p*-AKT (Cat no.: 4060S), and Lamin A/C (Cat no.: 4777T) were provided by Cell Signaling Technology, MA, USA; HO-1 (Cat no.: 10701-1-AP), Bcl2 (Cat no.: 26593-1-AP), and Bax(Cat no.: 50599-2-1g) were provided by Wuhan Sanying Biotechnology Co., Ltd., Wuhan, China. Nrf2 (Cat no.: ab34436-050) was provided by Hangzhou Lianke Biotechnology Co., Ltd., Hangzhou, China.

### Preparation of Extraction Parts of 
*Z. bungeanum*

The dried *Z. bungeanum* (100 g) was decocted two times (0.5 L each) with water, two filtrates were combined and concentrated to 100 ml (WEZ). The dried *Z. bungeanum* (200 g) was extracted with volatile oil extractor and afforded volatile oil 5.8 ml (VOZ). The dried *Z. bungeanum* (500 g) was extracted three times (5 L each) with petroleum ether (PEZ) and methylene chloride (MCZ), respectively, at room temperature and afforded a PEZ extract (33.31 g) and an MCZ extract (33.36 g).

### Animal Grouping and Administration

Three grams of the raw material per 60 kg human body was used to calculate the dosage following the standard of Chinese Pharmacopoeia 2015 Edition (Commission C.P., 2015). Then according to the ratio of mice to human body surface area of 1:9, the dose of mice was 450 mg/kg (Nair and Jacob, 2016). KM mice were randomly divided into seven groups: control group (distilled 0.5% CMC-Na orally and normal saline subcutaneously), model group (distilled 0.5% CMC-Na orally and 500 mg·kg ^-1^ D-galactose subcutaneously), hydergine group (0.9 mg·kg ^-1^ dihydroergotoxine mesylate tablets orally and 500 mg·kg ^-1^ D-galactose subcutaneously), WEZ group (water extract of 450 mg·kg ^-1^ *Z. bungeanum* orally and 500 mg·kg ^-1^ D-galactose subcutaneously), VOZ group (volatile oil of 450 mg·kg ^-1^ *Z. bungeanum* orally and 500 mg·kg ^-1^ D-galactose subcutaneously), PEZ group (petroleum ether extract of 450 mg·kg ^-1^ *Z. bungeanum* orally and 500 mg·kg ^-1^ D-galactose subcutaneously), and MCZ group (methylene chloride extract of 450 mg·kg ^-1^ *Z. bungeanum* orally and 500 mg·kg ^-1^ D-galactose subcutaneously) with 10 mice in each group, orally administered once daily. Half an hour after gavage, D-galactose or normal saline was injected subcutaneously once a day, continuously for 48 days. On the 48th day, tissue preparation was carried out after the animals were sacrificed by decapitation. The right hemisphere of five mice in each group was quickly fixed with 4% paraformaldehyde for 48 h before embedding in paraffin. The hippocampus was isolated on ice, then stored in liquid nitrogen.

### Passive Avoidance Test

The passive avoidance apparatus consists of an illuminated white compartment and a dark compartment with a movable door connected, and inescapable electrical shocks were delivered to the animals when they enter the dark chamber because the dark compartment was electrified. On the 40th day, mice were placed into the apparatus to familiarize with the surroundings for 5 min (Moosavi et al., 2018). Twenty-four hours after the first trial, the passive avoidance test was performed, the mice were placed into the bright compartment, opened the movable door, the latency time taken to enter the dark compartment was recorded as “escape latency,” and the number of times the mice entered the black box was counted as “the number of errors.” If the mouse did not enter into the dark chamber within 300 s, a latency of 300 s was recorded (Wei et al., 2019).

### Morris Water Maze Test

The mice were placed in EthoVision XT Morris water maze video tracking test system, which can automatically record time for mice to find platform. The training time was set as 60 s, if the mice could not reach the terminal point within 60 s was recorded as 60 s. Attentively, it needs to be artificially guided to stay on the platform for 10 s if the mice did not find the platform in the first 4 days. Mice were trained every 24 h, and experimental data were collected on the fifth day (Luo et al., 2014). On the sixth day, the platform was removed to test the spatial searching ability of the mice, following the number of mice passed through where the place was initially placed, and the time stayed in the destination quadrant were recorded (Vorhees and Williams, 2006).

### Hematoxylin–Eosin Staining and Nissl Staining

The right hemispheres of mice in each group were embedded in paraffin and cut into 4-µm slices, then stained with hematoxylin–eosin (HE) staining. The embedded wax pieces were cut into thick and dewaxed by xylene and gradient concentrations of ethanol. After rinsing with phosphate-buffered solution (PBS), it was stained with Nissl staining (Liang et al., 2019). The stained slices were observed and photographed under an optical microscope for examining the pathological changes and comparing number of Nissl bodies in the hippocampus. The damage score of neurons was assessed accordance to the scoring criteria by Shi et al. (2017). The Nissl body counts in hippocampus CA1 and the CA3 regions from each animal were calculated using ImageJ software (Liang et al., 2019).

### Measurement of the Oxidation State

The activity of SOD and CAT and the content of MDA and GSH were determined strictly according to the kit instructions.

### Western Blot

The hippocampal samples were removed from liquid nitrogen. The lysis buffer for ice cracking was added to the samples (1 mg:10 μl), then centrifuged at 12,000 × g to obtain the supernatant. Then, sodium dodecyl sulfate (SDS)-polyacrylamide gels electrophoresis was used to isolate target protein from total proteins. The proteins were transferred onto a polyvinylidene fluoride (PVDF) membrane at low temperature with a cold pack, and the membrane was blocked with the blocking buffer (containing 5% bovine serum albumin). After the blocking at room temperature was completed, the first antibodies were added onto the membrane respectively and which was incubated at 4°C overnight. Then, the membrane was washed with Tris-buffered saline with Tween 20 (TBST). The second antibody (1:5,000) was add onto the membrane and incubated for 90 min, then rinsed with TBST. Finally, the relative expression of target protein was analyzed by the Quantity One software.

### Statistical Analysis

Behavioral data are presented as mean ± standard error of the mean (SEM). Other data are presented as the mean ± standard deviation (SD). The results of escape latency in Morris water maze test were analyzed using two-way analysis of variance (ANOVA). Other data were analyzed by one-way ANOVA. Bonferroni *post hoc* analysis was conducted, and the statistical significance was tested at *P* < 0.05.

## Results

### Effects of the Different Extractions of *Z. bungeanum* on D-Galactose-Induced Cognitive Impairment

**Figure 2:**
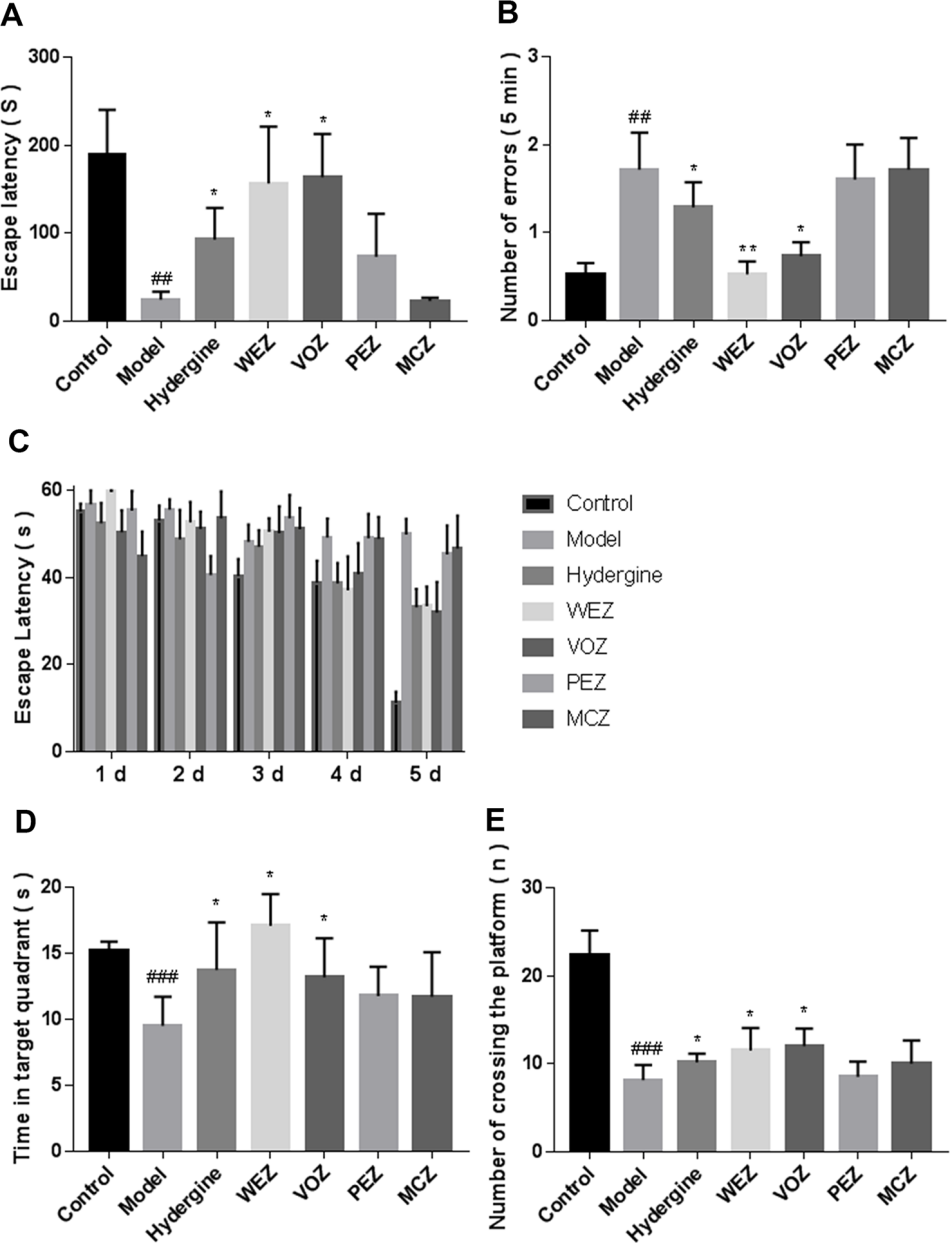
D-galactose induced cognitive impairment. Effects of *Z. bungeanum* extracts by water (WEZ), volatile oil (VOZ), petroleum ether (PEZ), methylene chloride (MCZ) on the escape latency **(A)** and the number of errors **(B)** within 5 min on the passive avoidance test of mice. Escape latency **(C)**, swimming time in the target quadrant **(D)**, and the number of passing the platform **(E)** in the Morris water maze during the five consecutive training trial days (mean ± SME, n = 10). ^*^ *P* < 0.05 or **P < 0.01 versus the model group, ^##^ *P* < 0.01, or ^###^ *P* < 0.001versus the control group. Comparison of escape latency was performed with two-way analysis of variance (ANOVA) in the Morris water maze during the five consecutive days training trial days, others were performed by one-way analysis of variance (ANOVA).

To evaluate whether different extractions of *Z. bungeanum* could ameliorate the memory dysfunction of D-galactose-induced aging mice, we conducted the passive avoidance test (**Figures 2A, B**). The escape latency of the model group was significantly shortened (*P* < 0.01), and change to the number of errors was increased (*P* < 0.01) in comparison with the control group, which indicated that the D-galactose-induced impaired retention of the passive avoidance test. Nevertheless, compared with the model group, the escape latencies of WEZ group and VOZ group were prolonged; moreover, the numbers of errors of the WEZ group and VOZ group were significantly decreased. While those of PEZ group and MCZ group did not show a significant difference from the model group (*P* > 0.05). These results showed that WEZ and VOZ extracts ameliorated the D-galactose-induced fear memory impairment.

Effects of different extractions of *Z. bungeanum* on the spatial and long-term memory of the D-galactose-induced mice were tested *via* Morris water maze. In the orientation navigation test, the latency of all groups to find the platform gradually was shortened [F (4, 20) = 30.59, *P* < 0.001] as training time progressed (**Figure 2C**). Moreover, the model group exhibited longer latency to reach the platform than the control group [F (4, 20) = 12.05, *P* < 0.001]; administration of WEZ [F (4, 50) = 2.923, *P* < 0.05] and VOZ [F (4, 20) = 2.582, *P* < 0.05] significantly attenuated the increased latency to reach the platform, while the mice treated with PEZ and MCZ extracts did not show significant reduction. The results of spatial search test are shown in **Figures 2D, E**, which manifested D-galactose could significantly decrease the swimming time spent in the target quadrant (*P* < 0.001) and the number of passing the platforms of model group (*P* < 0.001) in comparison to control group. The mice treated with WEZ and VOZ extracts lengthened the residence time in the target quadrant and enhanced the number of passing the platform zone compared with the model group (*P* < 0.05). Whereas the mice treated with PEZ and MCZ extracts could not lengthen the swimming time in the target quadrant and could not enhance the number of passing the platform zone compared with the model group. These results showed that in the mice treated with WEZ and VOZ extracts, the D-galactose-induced spatial and long-term memory dysfunction had been reduced.

### WEZ and VOZ Extracts Ameliorated D-Galactose-Induced Hippocampal Neuron Damage

**Figure 3:**
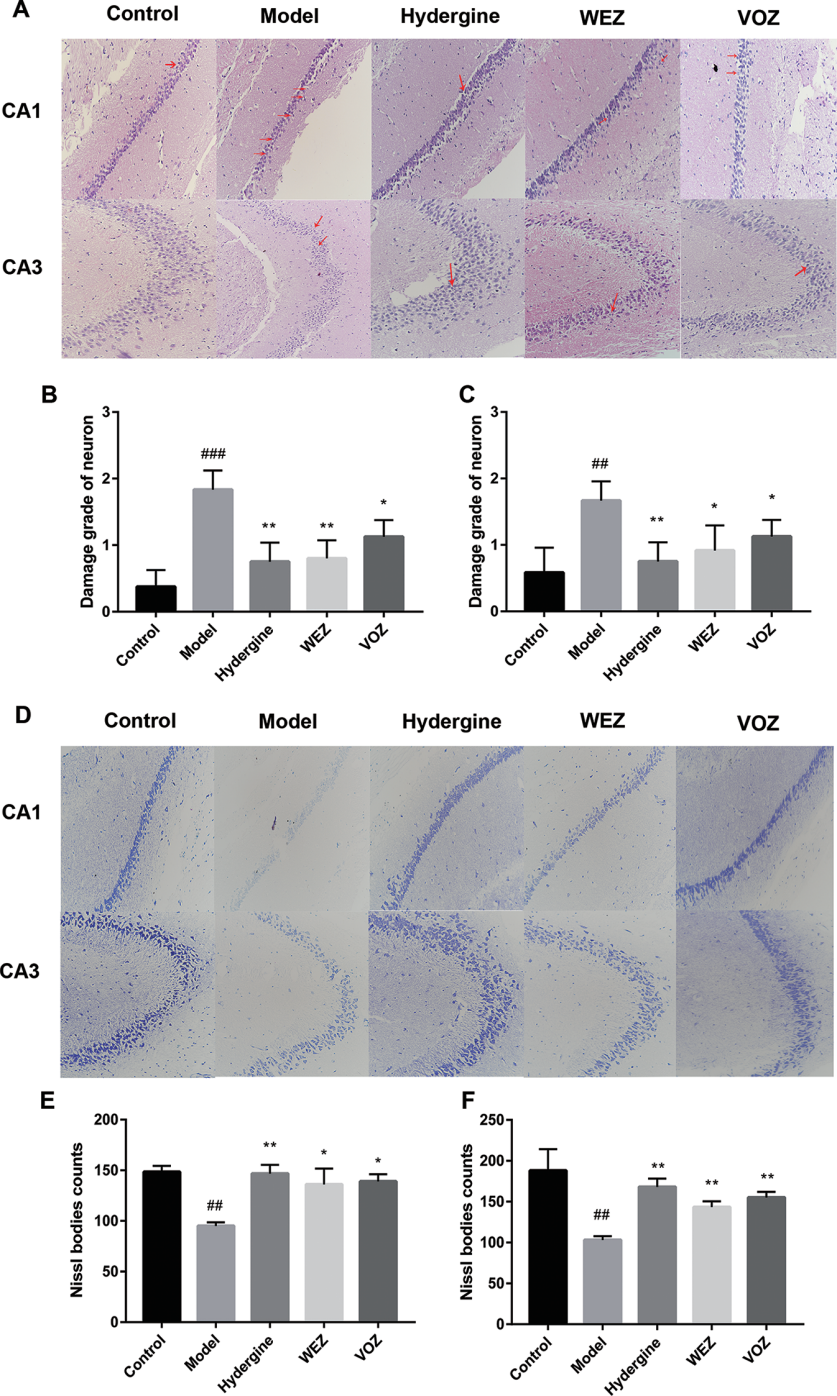
Effects of water (WEZ) and volatile oil (VOZ) on the D-galactose-induced pathological damage and Nissl bodies of hippocampus. **(A)** Representative pictures of hematoxylin–eosin (HE) staining showed WEZ and VOZ extracts attenuated the damage to the hippocampus in the D-galactose model, magnification: 200×. **(B)** The grade of neuronal damage in the CA1 region of D-galactose model mice (mean ± SD, n = 5). **(C)** The grade of neuronal damage in the CA3 region of D-galactose model mice (mean ± SD, n = 5). **(D)** Representative pictures of Nissl staining showed WEZ and VOZ extracts increased the number of hippocampal Nissl bodies in the D-galactose model, magnification: 200×. **(E)** The Nissl body counts in the CA1 region of D-galactose model mice (mean ± SD, n = 5). **(F)** The Nissl body counts in the CA3 region of D-galactose model mice (mean ± SD, n = 5). Red arrows indicate atrophy or loss of neurons. ^*^ *P* < 0.05 or **P < 0.01 compared with the model group, ^##^ *P* < 0.01 or ^###^ *P* < 0.001 compared with the control group. Comparisons were performed by one-way analysis of variance (ANOVA).

The ability of cognitive function is closely related to the structure of hippocampus. To further investigate the role of WEZ or VOZ extracts on cognitive dysfunction, we tested the effects of WEZ and VOZ extracts on the hippocampus pathology of the D-galactose-induced aging mice. The results of HE staining showed the hippocampus of control group had no remarkable abnormalities; hippocampal neurons were arranged neatly with no noticeable cell loss (**Figure 3A**). In contrast, atrophy and loss of neurons in the hippocampus were observed in the model group; meanwhile, the scores of neuron damage grade were markedly increased as compared with the control group (**Figures 3B, C**). While nerve cell damage and the scores of neuron damage grade were reduced in the CA1 and CA3 regions of mice after treatment with hydergine, WEZ, and VOZ extracts.

The results of Nissl staining showed that the Nissl bodies counts in hippocampal CA1 and CA3 of model group were prominently lower compared with the control group (**Figure 3D**). Whereas, the counts were higher compared with the model group after administration of hydergine, WEZ and VOZ extracts (**Figures 3E, F**). The results of HE staining and Nissl staining demonstrated that hippocampal histopathological alterations and neuronal loss induced by the D-galactose were alleviated after treatment with WEZ and VOZ extracts.

### Effects of WEZ and VOZ Extracts on Oxidative Status of Mouse Brain Induced by the D-Galactose

**Figure 4:**
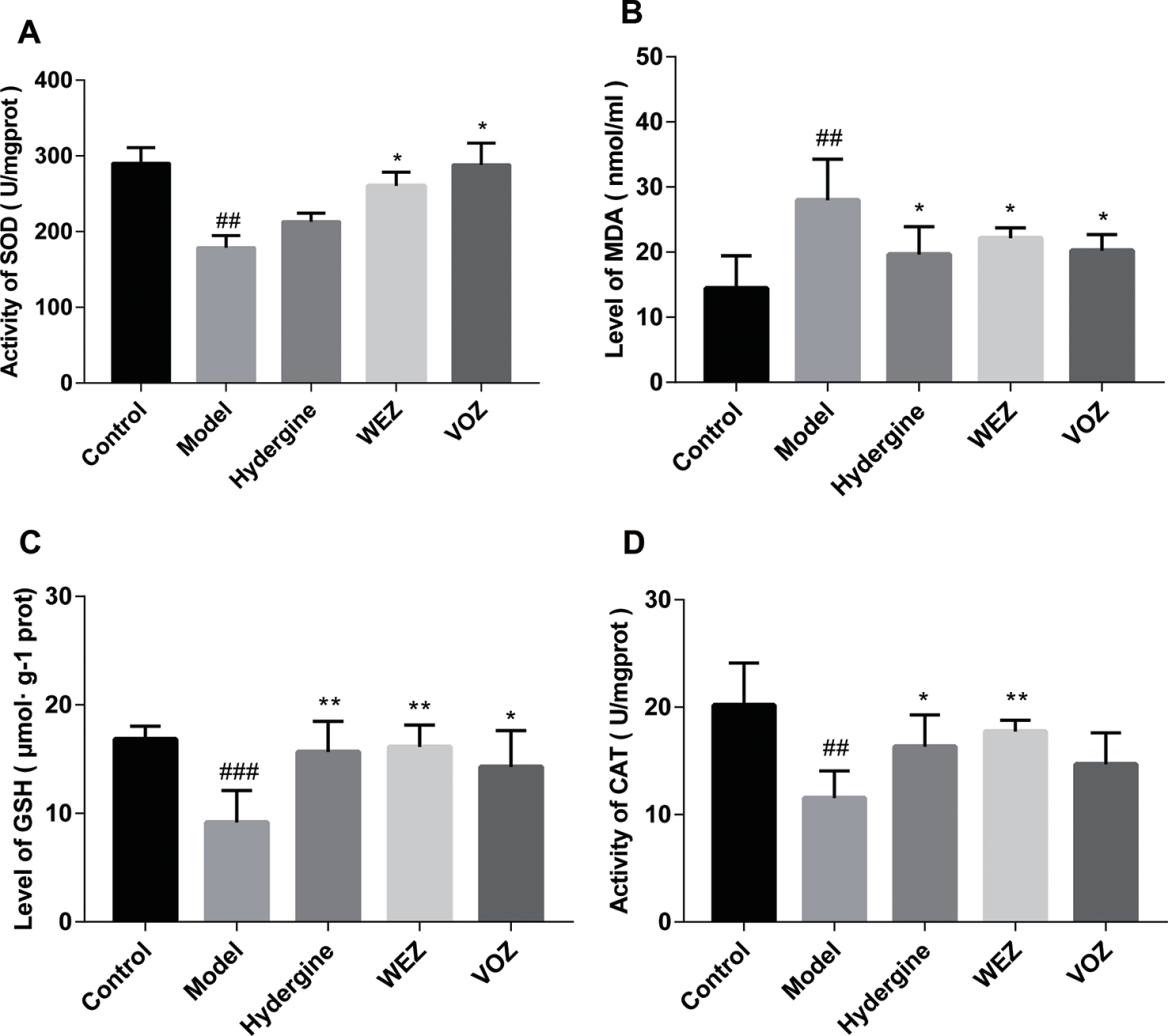
Effects of water (WEZ) and volatile oil (VOZ) extracts on oxidative status in the brain tissue of mice. Effects of WEZ and VOZ on superoxide dismutase (SOD) activities **(A)**, malondialdehyde (MDA) levels **(B)**, glutathione (GSH) levels **(C)**, catalase (CAT) activities **(D)** of D-galactose-induced mice (mean ± SD, n = 5). ^*^ *P* < 0.05 or **P < 0.01 compared with the model group, ^##^ *P* < 0.01 or ^###^ *P* < 0.001 compared with the control group. Comparisons were performed by one-way analysis of variance (ANOVA).

Tons of studies have proved that oxidative status may underlie premature aging in these animals, the activity of some crucial antioxidant enzymes including SOD and CAT, the levels of oxidation product MDA and non-enzymatic antioxidant GSH in the mouse brain were detected. As illustrated in **Figures 4A, B, D**, the activities of SOD and CAT and the level of GSH in the brain tissue were remarkably suppressed after exposure to D-galactose compared with the control group (*P* < 0.01 or *P* < 0.001). Nevertheless, the decrease in these antioxidant enzymes and biomolecule was noticeably ameliorated by WEZ and VOZ extract treatment (*P* < 0.05 or *P* < 0.01). As shown in **Figure 4C**, MDA in the brain tissue of mice in the model group was increased compared with the control group (*P* < 0.01). But WEZ and VOZ supplementation decreased the MDA levels in the brain compared with the model group (*P* < 0.05). This experiment showed that oral administration of WEZ and VOZ extracts could improve the redox balance of mouse brain tissue and then prevent the oxidant status induced by the D-galactose.

### WEZ and VOZ Extracts Activated PI3K/Akt/Nrf2 Axis in D-Galactose-Induced Aging Mice

**Figure 5:**
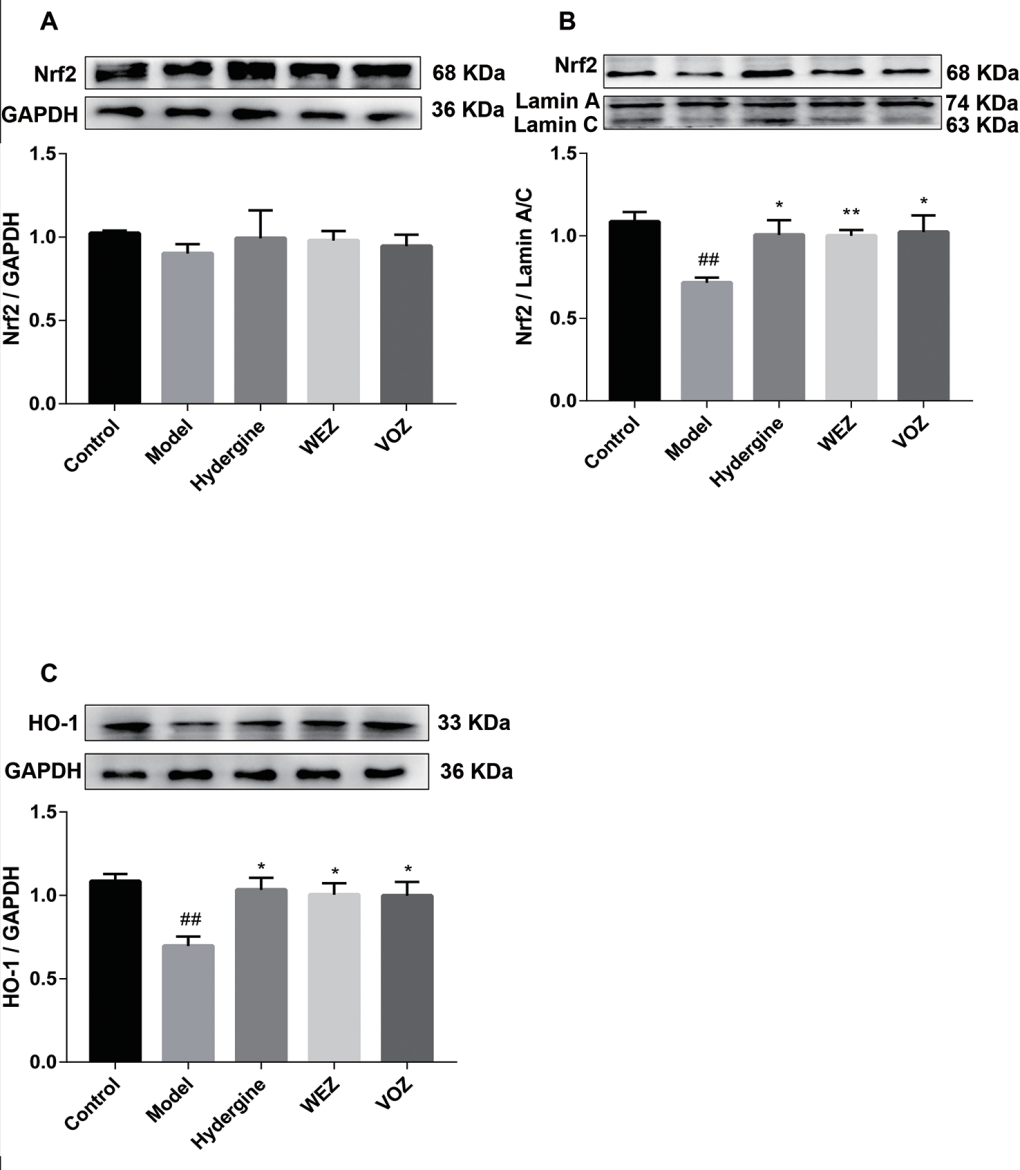
Water (WEZ) and volatile oil (VOZ) extracts activated nuclear factor erythroid 2-related factor 2 (Nrf2)/heme oxygenase 1 (HO-1) signaling pathway in D-galactose-induced aging mice. Effects of WEZ and VOZ extracts on the expression of Nrf2 in the cytoplasm **(A)**, expression of Nrf2 in the nucleus **(B)**, and the expression of HO-1 **(C)** (mean ± SD, n = 5). ^*^ *P* < 0.05 or **P < 0.01 compared with the model group, ^##^ *P* < 0.01 compared with the control group. Comparisons were performed by one-way analysis of variance (ANOVA).

Nrf2 plays an important role in participating in cellular antioxygen stress. And we have demonstrated WEZ and VOZ extracts could improve oxidation state of mouse brain tissue induced by the D-galactose. Hence, the expression of Nrf2 in the nucleus and cytoplasmic fraction were examined, respectively. The results (**Figures 5A, B**) displayed that the expression of Nrf2 in the D- galactose-induced aging mice was restrained in the nucleus and cytoplasmic fraction compared with the control group (*P *< 0.01 or *P *< 0.05). After treatment with WEZ and VOZ, the expression of Nrf2 was improved in the nucleus compared with the model group but WEZ and VOZ could not apparently change the expression of Nrf2 in the cytoplasm. In addition, the downstream protein HO-1 of Nrf2 was detected by Western blot. As shown in **Figure 5C**, the expression of HO-1 protein was reduced in aging mice compared with that in control mice (*P* < 0.01). But the expression level of HO-1 was increased after WEZ and VOZ treatment (*P* < 0.05). The results indicated that Nrf2 nuclear translocation is facilitated after treatment with WEZ and VOZ in D-galactose-induced mice.

**Figure 6:**
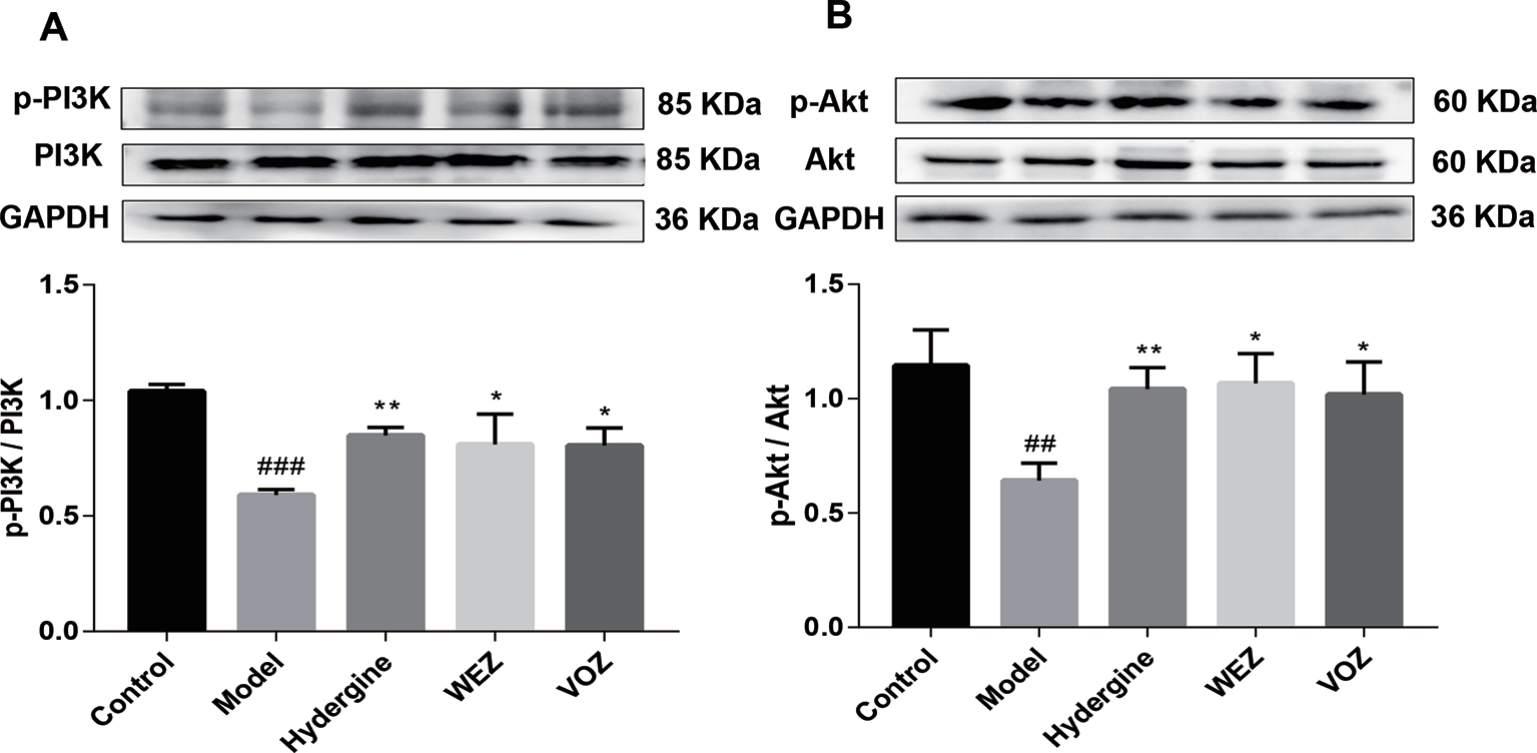
Water (WEZ) and volatile oil (VOZ) extracts activated phosphoinositide 3-kinase (PI3K)/protein kinase B (Akt) signaling pathway. The effects of WEZ and VOZ extracts on the expression ratio of *p*-PI3K/PI3K **(A)**, *p*-Akt/Akt **(B)** (mean ± SD, n = 5). ^*^ *P* < 0.05 or **P < 0.01 compared with the model group, ^##^ *P* < 0.01 or ^###^ *P* < 0.001 compared with the control group. Comparisons were performed by one-way analysis of variance (ANOVA).

According to research reports, activation of PI3K/AKT signaling pathway can promote Nrf2 transport from cytoplasm to nucleus (Nguyen et al., 2004). Therefore, PI3K/AKT signaling pathway was tested (**Figure 6**). Compared with the control group, the protein levels of PI3K phosphorylation and Akt phosphorylation were significantly reduced in model group (*P* < 0.01 or *P* < 0.001). However, phosphorylation levels of PI3K and Akt were significantly upregulated after WEZ and VOZ treatment. The findings imply that WEZ and VOZ could activate Nrf2 to prevent the D-galactose-induced oxidative stress that may be regulated by the PI3K/AKT signaling pathway. Given the findings mentioned above, we speculated that the protective effect of WEZ and VOZ extracts might be mediated through the activation of PI3K/Akt/Nrf2 signaling pathway.

### WEZ and VOZ Extracts Suppressed Apoptosis Induced by D-Galactose

**Figure 7:**
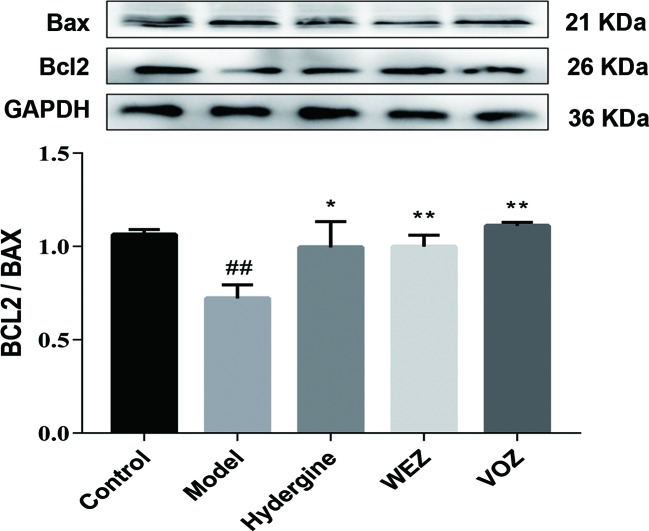
Water (WEZ) and volatile oil (VOZ) extracts suppressed the apoptosis of hippocampal neurons induced by D-galactose in mice. Effects of WEZ and VOZ extracts on the expression ratio of Bcl2/Bax (mean ± SD, n = 5). ^*^ *P* < 0.05 or **P < 0.01 compared with the model group, ^##^ *P* < 0.01 compared with the control group. Comparisons were performed by one-way analysis of variance (ANOVA).

In order to investigate effects of WEZ and VOZ extracts on cell apoptosis in hippocampus next, we had evaluated another hallmark of neuronal cell loss—apoptosis. According to our findings, there was enhanced expression of B-cell lymphoma 2 (Bcl2)-associated X, apoptosis regulator (Bax) and reduced expression of (Bcl2) in the D-galactose-induced mice. Interestingly these markers were markedly reversed in the WEZ and VOZ groups (**Figure 7**). And WEZ and VOZ significantly elevated the expression ratio of Bcl2/Bax in D-galactose-induced mice (*P* < 0.01 or *P* < 0.05).

## Discussion

The present study provides support for investigation of Nrf2 pathway activation by *Z. bungeanum* as a novel strategy to ameliorate behavioral dysfunction and neurological deficits in a D-galactose-induced aging mouse model. We found that WEZ and VOZ extracts protected against cognitive dysfunction and hippocampal neuronal cell damage induced by D-galactose. In addition, our results also indicated that WEZ and VOZ extracts could activate PI3K/Akt/Nrf2 pathway to exert antioxidant, antiapoptosis, and anti-neurodegenerative effects. Therefore, *Z. bungeanum* might have potential effects for further development as an effective natural herbal product in antiaging and its associated diseases. Interestingly, this is the first time to demonstrate the effect and mechanism underlying of *Z. bungeanum* on the learning and memory impairment in the D-galactose-induced mouse model.

*Z. bungeanum* is often used as a flavor condiment and traditional herbal medicine, which has shown benefit effects on neuronal impairment. For example, compound Daikenchuto, which is a component of *Z. bungeanum* fruit, could improve cognitive deficits of mice on the scopolamine-induced dementia (Nakamura et al., 2006). Isobutyl hydroxyamides from Sichuan pepper showed protective activity on PC12 cells damaged by corticosterone (Chen et al., 2018). Another example, Gx-50, isolated from *Z. bungeanum* could reduce neuronal calcium toxicity and enhance cognitive function of Aβ protein precursor transgenic (Tang et al., 2013). It has been reported that the water and ethanol extract of *Z. bungeanum* contains polyphenols, which can remove MDA, 1,1-diphenyl-2-picryhydrazyl (DPPH), hydroxyl radical (·OH), and nitrite, and the volatile oil of *Z. bungeanum*, and also has certain scavenging ability to DPPH free radicals (Mi et al., 2004; Li et al., 2015). Oxidative stress has been considered a key mediator to induce neuronal degeneration in various age-associated neurodegenerative diseases such as AD (Ali et al., 2015). We speculated that *Z. bungeanum* with antioxidant properties may be able to protect against neurodegenerative diseases.

D-galactose-induced mammalian aging model is commonly used in cognitive and neurological dysfunction because a series of neurobehavioral and neurochemical studies in rodents showed D-galactose injection induced cognitive disorder, increased free radical production, decreased antioxidant enzyme activity, and reduced immune response (Sadigheteghad et al., 2017). But the effect of *Z. bungeanum* on cognitive impairment and neurological dysfunction induced by D-galactose has not been reported before. Here, we investigated effects of *Z. bungeanum* extractions on cognition impairment of D-galactose-induced aging mice. The results showed that WEZ and VOZ extracts can prevent the D-galactose-induced cognitive impairment and neurodegeneration. And our study illuminated that WEZ and VOZ extracts could significantly reduce the level of MDA, increase the content of GSH, and improve SOD and CAT activity in the brain tissue of mice induced by D-galactose. Therefore, it is reasonable to suspect that oxidation regulator plays an important role in the neuroprotective effect of WEZ and VOZ extracts.

It is reported that Nrf2 pathway is an important target in the treatment of neurodegenerative diseases (Wu et al., 2015). Under normal physiological conditions, Nrf2 binds to Keap1, a cysteine-rich protein, located in the cytoplasm. However, when exposed to oxidative conditions, Keap1 denatures, and Nrf2 translocates into the nucleus, binds to the antioxidant elements (AREs), and then upregulates expression of antioxidant enzyme genes, such as SOD and CAT (Nguyen et al., 2003). Studies have reported that the decreased of Nrf2 nuclear translocation in natural aging animals (Tomobe et al., 2012). Consistent with previous studies, the study also showed that the Nrf2 translocation to the nucleus was inhibited by the D-galactose treatment (Zhang et al., 2017), and treatment with WEZ and VOZ extracts had shown effective promotion. HO-1 is an important antioxidant enzyme, which can exert neuroprotective effect by antioxidant (Zhao et al., 2018). Meanwhile, after treated with WEZ and VOZ, Nrf2 nuclear accumulation gave rise to promoting the expression of HO-1, a downstream protein of Nrf2, which is not only confirmed the involvement of Nrf2, but indicated the central role of HO-1 protein in the antioxidant protection of WEZ and VOZ extracts. Therefore, WEZ and VOZ extracts, which can activate Nrf2 activator, could be promising for brain injury prevention during the aging process.

PI3K/Akt signaling pathway is involved in the regulation of cell proliferation, metabolism, growth, differentiation, apoptosis, and other life phenomena (Abeyrathna and Su, 2015). PI3K/Akt signaling is an important upstream pathway of Nrf2 (Nguyen et al., 2004). The activation of PI3K/Akt signaling pathway promotes the dissociation of Nrf2 and Keap1, thereby regulating the expression of Nrf2 in nucleus (Zhang et al., 2019). Numerous studies hinted that the PI3K/Akt signaling pathway protects against hyperoxia-induced acute lung injury by modulating Nrf2 (Reddy et al., 2015). Additionally, naringenin improved behavioral dysfunction and neurological deficits in D-galactose-induced aging mouse model *via* activation of PI3K/Akt/Nrf2 pathway (Zhang et al., 2017). Results of our work demonstrated that phosphorylation levels of PI3K and Akt were significantly upregulated after WEZ and VOZ treatment, which revealed that WEZ and VOZ extracts can activate PI3K/Akt signaling pathway to promote Nrf2 nuclear translocation preventing D-galactose-induced oxidative stress.

Apoptosis is a main player in neuronal cell loss, so here we examined Bax and Bcl2. According to our Western blot studies, there were enhanced expression of Bax and reduced expression of Bcl2 in the D-galactose model group. Interestingly, Bax was significantly reduced, while the level of Bcl2 was significantly upregulated with the administration of WEZ and VOZ extracts. *Z. bungeanum* modulatory effects against apoptosis have already been reported (Pang et al., 2019). As reported previously, suppression of apoptotic cell death may be partly due to the inhibition of oxidative stress (Deng et al., 2019).

AD is associated with aging, which is a progressive neurodegenerative disorder characterized by increasing deterioration of memory and cognition (Wu et al., 2019). Markers of oxidative damage are increased, for example, Nrf2 expression is decreased in the brains of AD, as well as in amyloid precursor protein (APP)/presenilin 1 (PS1) mutant mouse models of AD (Ramsey et al., 2007). Conversely, Nrf2 overexpression protects against toxicity induced by the Aβ42 peptide in mammalian cells and prevents neuronal pathology in mouse models of AD (Kanninen et al., 2009). Activation of Nrf2 is, therefore, increasingly regarded as an attractive target for the prevention of AD (Kerr et al., 2017). Consequently, it is possible that *Z. bungeanum* might work well in the treatment of AD patients, which is what we will study next.

## Conclusion

In summary, this study is the first to demonstrate that WEZ and VOZ extracts, not the PEZ or MCZ extracts, of *Z. bungeanum* could enhance the capacity of the inhibition of apoptosis and oxidative stress through activation of PI3K/Akt/Nrf2 axis, thereby easing cognitive dysfunction and neurological deficits in the D-galactose-induced aging mouse model. However, participation substances and whether other pathways in the process deserve further study.

## Data Availability Statement

The datasets analyzed in this article are not publicly available. Requests to access the datasets should be directed to xushijun@cdutcm.edu.cn.

## Ethics Statement

The animal study was reviewed and approved by The Ethics Committee for Animal Experiments of the Institute of Material Medical Integration and Transformation for Brain Disorders in Chengdu University of Chinese Medicine.

## Author Contributions

SX contributed to the conception and design of the study. MZ and PX performed the methodology and the practical work. XT did the data curation. DG completed hematoxylin–eosin (HE) staining and Nissl body staining. MZ also did the writing of the original draft. FW and SX modified the manuscript.

## Funding

This research was supported by the Key Research and Development Plan of Sichuan Province (No. 19ZDYF0600) and the Science and Technology Program of Sichuan Province (No. 18ZDYF1175).

## Conflict of Interest

The authors declare that the research was conducted in the absence of any commercial or financial relationships that could be construed as a potential conflict of interest.
